# Current progress of skin tissue engineering: Seed cells, bioscaffolds, and construction strategies

**DOI:** 10.4103/2321-3868.118928

**Published:** 2013-09-18

**Authors:** Huanjing Bi, Yan Jin

**Affiliations:** 1 Xi’an Institute of Tissue Engineering and Regenerative Medicine, Xi’an, Shaanxi, China; 2 Research and Development Center for Tissue Engineering, College of Stomatology, Fourth Military Medical University, Xi’an, Shaanxi, China; 3 Research and Development Center for Tissue Engineering, College of Stomatology, Fourth Military Medical University, Xi’an, Shaanxi, 710032 China

**Keywords:** Regenerative medicine, wound healing, biomaterials, seed cells, tissue-engineered skin

## Abstract

The development of cell biology, molecular biology, and material science, has been propelling biomimic tissue-engineered skins to become more sophisticated in scientificity and more simplified in practicality. In order to improve the safety, durability, elasticity, biocompatibility, and clinical efficacy of tissue-engineered skin, several powerful seed cells have already found their application in wound repair, and a variety of bioactive scaff olds have been discovered to influence cell fate in epidermogenesis. These exuberant interests provide insights into advanced construction strategies for complex skin mimics. Based on these exciting developments, a complete full-thickness tissue-engineered skin is likely to be generated.

## Introduction

**Figure 1:**
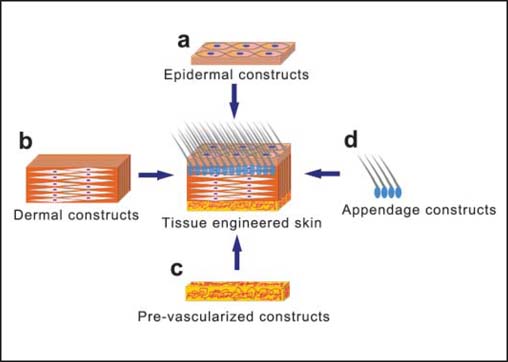
A schematic diagram of a desirable full-thickness TES. (a) The relatively thin keratinized epidermal layer provides protective function that prevents infection and fluid loss. (b) A much thicker dermal layer underlies the upper layer and consists of abundant mesenchymal cells and ECM proteins, forming the basal body of TES. (c) To increase the survival of TES, a well-vascularized layer containing nerve receptors should be integrated under the dermal capillary network. (d) Skin appendages including hair follicles and sweat glands embedded in the dermal layer are also indispensable parts of full-thickness TES that best mimic the function as well as appearance of normal skin.

As the largest organ, skin covers the entire exterior of the body and takes over about 8% of the total body mass. Due to its direct exposure to potentially harmful microbial, thermal, mechanical, and chemical damages, the loss of skin can occur for many reasons, including disorders, acute trauma, chronic wounds, or even surgical interventions.[1] Tissue-engineered skin (TES) substitutes represent a logical therapeutic option for the treatment of acute and chronic skin wounds. Since the successful isolation and cultivation of human epidermal keratinocytes in 1975,[2] TES has developed from epidermal substitutes to full-thickness skin containing different seed cells [Figure 1]. According to their anatomical structures, skin substitute products could be classified into cellular epithelial autografts, engineered dermal substitutes, and engineered dermo-epidermal composite substitutes.[3] All these products have become a prospective measure for clinical treatment of large full-thickness skin defects.

Recently, highly innovative progress in the development and clinical use of highly sophisticated TES in order to increase their medical safety, durability, elasticity, biocompatibility, and clinical efficacy has been reported. Here, we give a brief introduction to some representative progress in feasible seed cells and supportive biomaterials, as well as their applications in varied TES substitutes in developing our understanding of skin tissue engineering.

**Table:** 

Access this article online	
**Quick Response Code:**	**Website:** www.burnstrauma.com
	**DOI:** 10.4103/2321-3868.118928

## Seed cells for skin tissue engineering

### Skin-derived seed cells

#### Keratinocytes

Up to 1980s, the development of *in vitro* expansion technique unveils the unlimited proliferation capacity of keratinocytes that, they are capable of being passaged for many hundreds of generations without undergoing senescence.[4] Such exciting characteristics make keratinocyte an ideal alternative treatment for large burns, instead of skin grafting. Since then, numerous clinical trials have demonstrated the efficacious application of cultured keratinocytes in wound healing.[5,6] As the first step of treatment with skin substitutes, single cell suspension and multilayered “keratinocyte sheet” were explored.[7,8] However, due to lack of dermal structures, simple keratinocyte coverage is fragile and the take rate varies depending on the condition of the defected area. These major disadvantages make keratinocytes not optimal for the coverage of full-thickness defects [Table 1]. To further facilitate cell survival and growth, several bioscaffolds were comprised into the keratinocytes containing TES, such as collagen,[9] gelatin,[10] fibrin,[11] chitosan,[12] and various synthetic materials.[13–15] Moreover, in order to overcome some major drawbacks like scar formation and contracture, more dermal lineage seed cells need to be utilized to support the tissue-engineered constructs.

**Table 1:** Summary of available seed cells in TES constructs

Source	Potential usages	Autologous possibility	Identified isolation	Unlimited propagation	Pluripotency	Immunogenicity	Tumorigenicity	Legal restriction	Reference
**Skin-derived seed cells**									
Keratinocytes	E	•	•	•*		•			[4–6]
Dermal fibroblasts	D	•	•			•			[17, 19]
Epidermal stem cells	E/D	•		•	•	•			[20, 22, 25, 26]
Melanocytes	E	•	•			•			[30]
**Non-cutaneous cells**									
Embryonic stem cells	E/D		•	•*	•			•	[32–34, 36]
Inducible pluripotent stem cells	E/D	•	•	•*	•	•	•	•	[32, 40–42]
Mesenchymal stem cells	E/D	•	•		•				[47, 48, 50, 51]
Endothelial cells	D	•	•			•			[58, 59]
Amniotic cells	E/D				•				[63, 64]

E, epidermis; D, dermis; E/D, epidermis or dermis; •, reported character; *, feeder cell needed

#### Dermal fibroblasts

As the major cell type present in the dermis, fibroblast plays an important role in the wound healing process by producing extracellular matrix (ECM) proteins and cytokines. It has been shown that the presence of fibroblasts in three-dimensional (3D) constructs could significantly increase the survival[16] and proliferation[17] rate of keratinocytes. In a bilayered model, the underlying fibroblast promoted the keratinocyte layer into basal, granular, and cornified layers, in addition to promoting keratinocyte proliferation.[18] In addition, the synthesis and deposition collagen and other ECM components form the basis of the granulation tissue, which is a critical process in wound healing. Besides, in response to site-specific microenvironment, fibroblasts from undamaged tissue are able to migrate to the wound and transdifferentiate into myofibroblasts that take on a contractile phenotype.[19] Such characteristics make the fibroblasts a popular candidate for skin substitutes, especially those containing dermal equivalents.

#### Epidermal stem cells

Epidermal stem cells are a population of adult somatic stem cells specifically located in the basal cell layer of the epidermis. In addition to their low tendency to divide, their extensive and sustained self-renewal capacity plays an essential role in the lifelong epidermogenesis and wound repair of the skin.[20,21] Studies have shown that epidermal stem cells can be inducted into epidermis and its appendages such as sweat gland, hair follicle, and sebaceous follicle under certain microenvironments.[22–24] However, one major technical challenge that hampers the wide application of epidermal stem cells in skin tissue engineering is lack of specific surface antigens for their isolation or enrichment. To overcome this problem, several methods have been used to identify and differentiate them from other types of cells residing in the epidermis.[4,25,26] Since most epidermal stem cells were discovered from the “bulge” region of the hair follicle,[22,27] it is logically predicable to find out that epidermal stem cells have the ability to rebuild skin appendages such as hair follicle and sweat gland when applied in skin tissue engineering.[28] Although the regulation of epidermal stem cell differentiation is not clearly understood, these researches provide hope that functional skin substitutes equipped with appendages can be generated for transplantation in, for example, cases of extensive burns.

#### Melanocytes

Melanocytes are found in the lower layer of the epidermis and provide pigments to determine skin color. Theoretically, they are ideal seed cells to resolve the lacking of natural skin pigmentation which is one major esthetic concern in the current cultured skin grafts. *In vitro* reconstruction of keratinocytes combined with melanocytes on de-epidermized human dermis could result in a colored equivalent to natural epidermis.[29] Our previous work also revealed the feasibility of melanocytes in skin tissue engineering. After transplantation of constructed skin containing keratinocytes, melanocytes, and dermal fibroblasts, athymic mice generated black skins within 6 weeks.[30] Interestingly, *in vivo* research conducted by Hachiya *et al.* found that melanocytes are capable to spontaneously localize to the basal layer from a mixed cell slurry containing keratinocytes, fibroblasts, and melanocytes. In addition, the synthesized melanosome could be correctly transferred to keratinocytes after ultraviolet irradiation.[31] However, functional pigmentation is only the beginning, advanced method on controlling the production of appropriate amount of melanin deserves further investigation, in order to make TES visually indistinguishable with its surrounding tissue.

### Non-cutaneous cells

#### Embryonic stem cells

Embryonic stem cells (ESCs) are referred as pluripotent cells derived from inner cell mass of a blastocyst. With non-senescing self-renewal and differentiation capability into three germ layers, ESCs might offer a way to meet the challenge of biomimetic complex skin constructs. To initiate epidermal differentiation of ESCs, several protocols have been developed.[32–34] Besides, the transcriptional similarity of ESC-derived keratinocyte lineage and primary foreskin keratinocytes has been proved by microarray analysis, suggesting these cell types respond to specific microenvironment in a similar manner.[35] When grown in specific media and substrate conditions, ectodermal and mesenchymal cells derived from ESCs are capable of generating 3D epithelial tissue.[36] In a preclinical study, ESC-derived keratinocytes formed a pluristratified epidermis when seeded on an artificial matrix and finally regenerated a fully functional mature skin-like structure after *in vivo* implantation.[37] These studies provide a theoretical basis for the use of ESCs as seed cells for TES. However, embryonic stem cell research is handicapped by moral and ethical restraints, and the source of ESCs is thus problematic.[38]

#### Inducible pluripotent stem cells

In 2006, inducible pluripotent stem cells (iPSCs) were first discovered by Yamanaka and colleagues, who received The Nobel Prize in Physiology or Medicine in 2012 (jointly with John B. Gurdon) for the finding that mouse fibroblasts could acquire properties similar to those of ESCs after “reprogramming.”[39,40] Since this milestone, iPSCs have become a promising new source of stem cells for skin tissue engineering without any moral and ethical controversies. Recent publications show that procedures differentiating mouse iPSCs into epidermal keratinocytes are similar to keratinocyte differentiation of ESCs.[32,41–43] By using the iPSCs-derived keratinocyte lineage cells, Bilousova *et al.*, have successfully regenerated epidermis, hair follicles, and sebaceous glands in the skin of athymic nude mouse.[41] Though the exciting development has been achieved, utility of iPSCs for medical applications is still pending because of the possibility that the transgene technology may cause carcinogenesis and tumor formation.[44] As novel technologies relating to iPSCs are rapidly being developed,[45] the therapeutic applicability of iPSCs in skin tissue engineering and regenerative medicine will eventually be a reality.[46]

#### Mesenchymal stem cells

Mesenchymal stem cells are identified from functional tissues after birth (such as bone marrow, adipose, blood, and umbilical cord) and differ from ESCs in that they are more restricted in their proliferation and differentiation potential and cannot spontaneously give rise to complex tissues *in vitro*.[47] Among these, bone marrow mesenchymal stem cells (BMSCs) and adipose-derived stem cells (ADSCs) are perhaps the most promising candidates for skin regeneration.[48] The potential of such MSCs to differentiate into dermal cells has been described.[49,50] However, unlike many encouraging publications in the past decade that the differentiation of BMSCs across germinal boundaries is crucial for regeneration of injured tissues, recent reports suggest that the growth factors secreted by BMSCs are more important in stimulating proliferation and maturation of endogenous stem cell-like progenitors and decreasing inflammatory reactions.[51,52] Except for the trophic and immunomodulatory functions, BMSCs demonstrated abundant ECM remodeling, matrix contraction, and migration capability when cultured on dermal equivalents.[53,54] Evidences as our work showed that MSCs containing skin equivalent exhibited better healing and keratinization, less wound contraction, and more vascularization in a porcine deep partial-thickness burn wound model.[55] One study of a murine model of full-thickness defect treated with a biograft composed of ADSCs showed increased rate of re-epithelialization and vascularization.[56] Our preliminary work researched the vascularization capacities of two different scaffolds seeded with ADSCs — decellularized small intestinal submucosa (SIS) and acellular dermal matrix (ADM) — and found that both the ADSCs combining biomaterials revealed enhanced angiogenesis and wound healing rate compared with the non-seeded scaffolds.[57] All these findings strongly demonstrate that adult BMSCs/ADSCs can contribute to the healing of injured skin and might provide a cell resource for building TES.

#### Endothelial cells

Delivering cells of endothelial origin remains the most systematic approach to enhance neovascularization in TES equivalents, as these cells are destined to directly contribute to vessel formation.[58,59] Moreover, interactions between different seed cells with endothelial cells (ECs) may contribute to vascularization in TES through angiogenesis and/or vasculogenesis. By co-culturing dermal fibroblasts and ECs in collagen sponge *in vitro*, a complex vascular network with branching structures was formed.[60] In subsequent research using skin fibroblasts and ECs, Kunz-Schughart *et al.* verified that fibroblast could interact and support the migration, viability, and network formation of ECs.[61] Our result demonstrates an obvious implication of this work that a scaffold-free bilayered TES containing dermal fibroblasts together with dermal microvascular ECs could form capillary-like structures after 20 days of culture. Also, this vascularization process is associated with interactions among keratinocytes, fibroblasts, and ECs.[62] To further facilitate the clinical use of ECs, however, researches on overcoming their disadvantages like limited *in vitro* expansion capacity and potent immunogenicity are desiderated.

#### Amniotic cells

Due to the easy-to-gain and layered pattern, human amniotic membrane (AM) has a long history in skin wound healing.[63] Except for its mechanical support and protection, the epithelial regeneration and immunosuppressive potential of cellular components (namely, amniotic epithelial cells and amniotic mesenchymal cells) in AM have received much attention recently[64] For the first time, our previous research has shown the feasibility of both amniotic epithelial cells and amniotic mesenchymal cells in reconstruction of bilayered skin equivalent and thereafter skin regeneration.[65] Besides, recent work indicates that amniotic fluid-derived cells could act as a substitute seed cell for fibroblast in tissue-engineered dermo-epidermal skin analog.[66] Taken together, these findings suggest amnion might be a new source for obtaining the seed cells to develop new skin products for clinical application.

## Bioscaffolds in skin tissue engineering

The selection and optimization of a biomaterial scaffold plays a pivotal role in skin tissue engineering. Except for their mechanically supportive function, efficacious biocompatible scaffolds should assist the successful engraftment of TES, as they expediently accrete wound and promote granulation tissue formation, fibroblast-driven remodeling, angiogenesis, and re-epithelialization.[67] However, novel concepts of skin tissue engineering extend new and specific requirements for biomimetic scaffolds,[68] based on which many interesting natural and synthetic polymers have been used in constructing artificial skin.

### Natural polymers

#### Collagenous biomaterials

As a major ECM protein of the dermal layer of the skin, collagen is the most widely studied and clinically utilized natural scaffold available for TES substitutes. The advantages include good biocompatibility, proper porous structure, as well as low immunogenicity.[55,67,69] However, the poor mechanical strength and rapid biodegradation rate of natural collagen scaffolds limiting the graft instability are the critical disadvantages that hamper its applications. Therefore, to control the degradation, numerous works have focused on the mechanical properties of collagen, such as chemical and biophysical cross-linking techniques[70,71] or a structural modification method like dense film.[72] For example, addition of matrix protein tropoelastin to type I collagen enhanced the proliferation and migration rates of dermal fibroblasts *in vitro*.[73] Cross-linking collagen with chitosan after electrospinning resulted in a good potential for keratinocyte migration and wound re-epithelialization.[15] Addition of fibroblast growth factor 2 (FGF-2) or vascular endothelial growth factor (VEGF) to heparin cross-linked collagen scaffold increased its angiogenic potential.[74] Although effective modification and improvement have been made, other discouraging problems in collagen-based polymers for skin tissue engineering still exist, including the high cost of purification process, potential viral and prior contamination,[75] and variability in the physicochemical properties depending on source and processing.[76]

#### Noncollagenous biomaterials

Except for collagen, some other natural polymers have been investigated as scaffolds for skin tissue engineering, including gelatin, hyaluronic acid, fibrin, laminin, and elastin. Under various process conditions, these proteinic polymers could form different phases such as suspensions,[77] gels,[78,79] sponges,[80] films, or sheets.[81] Though these naturally derived molecules have been considered advantageous in their cell interaction and signaling contributions, the mechanical properties of these materials are often poor in comparison to the properties of synthetic materials.[82] Therefore, the noncollagenous biomaterials, like collagen itself, are often organized into synthetic matrices or to retain their stability and mimic the natural ECM.

#### Acellular matrices

ADM, which is derived from full-thickness skin by removing cells and cellular components rather than native dermal structure and extracellular proteins, has been successfully used both in pre-clinical animal studies and in human clinical applications.[83,84] Retaining structural and functional proteins that constitute the ECM, including collagen, fibronectin, laminin, and vimentin,[85] ADM provides an intrinsic microenvironment for cell adhesion and proliferation. Previous studies demonstrate that endogenous growth factors such as VEGF, FGF-2, and transforming growth factor β1 remain on ADM and are released into the surrounding tissue to accelerate processes such as angiogenesis, cell recruitment, cell division, and even potential antimicrobial activity.[86,87] Such biological benefits have led to the application of ADM-based skin substitutes in burn coverage for decades.[88,89] Besides its natural structures, our results show that even micronized ADM still facilitates cell adhesion and growth *in vitro* as well as forms a thick layered tissue when transplanted into subcutaneous muscle.[90]

### Synthesized materials

Compared to biological biomaterials, artificially synthesized polymers such as polyurethane (PU), polypropylene (PP),[91] polyglycolic acid (PGA), polylactic acid (PLA), and their copolymer poly (lactic-co-glycolic acid) (PLGA) display controllable mechanical properties and diverse plasticity.[92] It has been proved that PLGA-knitted matrix exhibits sufficient internal space for tissue ingrowth, in addition to its skeletal role in enhancing natural biopolymers like collagen.[93,94] Other polymeric biomaterials like polyvinyl alcohol (PVA) are capable of increasing structural stability and tensile strength, and improving initial cell proliferation when blended with collagen.[95] Unfavorable cell adhesion materials like poly (dl-lactide) (PDLLA) could interact and integrate well with dermal fibroblasts after addition of 30% poly (ethylene glycol) (PEG) before electrospinning.[96] Polyhydroxybutyrate (PHB) combines with organic-soluble chitosan and also reveals beneficial effect on promoting cell attachment and proliferation.[97] The progress in controlling scaffold property is exciting, but most of the current studies just demonstrate novel materials or methods without making comparison with any other available TES constructs. Predictably, but disappointingly, these works accumulate limited knowledge about the materials and their properties that are most suitable for TES.

To differentiate natural and artificial biomaterials from one another in only one specific aspect, several arrays screening of biomaterial have been developed.[98–100] These efforts provide *in vitro* pre-screening data for the selection of appropriate set of matrix molecules for skin tissue engineering. However, due to the complexity of *in vivo* microenvironment, more works such as testing different types of cells and additional modifications need to be extended in order to increase the possibility to successfully predict optimal biomaterials for skin tissue engineering.

## Current strategies for TES construction

With various approaches presently being developed in different laboratories and companies, a number of artificial skin equivalents are commercially available and many others are under development.[101] According to the complexity of their anatomical structures, the construction of TES could be classified from single-layered ones to ones with more than two layers.

### Single-layered substitutes

#### Epidermal substitutes

Based on the achievements made in *in vitro* cultivation and expansion of human keratinocytes, multiple autologous epidermal substitutes have been developed for re-epithelialization either by cell suspension (e.g. CellSpray[102]), subconfluent layer (e.g. Myskin[103]) or by sheet (e.g. Epicel,[104] EpiDex,[105] and EPIBASE[106]). The application of epidermal substitutes directly mitigates the deficiency of skin biopsy. However, the clinical integration of such cultured epithelial autografts is unpredictable and mostly relies on the condition of the wound bed, rather than the terminally differentiated keratinocytes.[1] Besides, the poor mechanical property and development of contractures due to the lack of dermis are also problematic to the epidermal substitutes.[107] This resulted in further requirements concerning the development of dermal substitutes.

#### Dermal substitutes

The mechanical stability and elasticity provided by dermal layer could prevent wound contraction and scar hyperplasia. In case of full-thickness burns, both dermal and epidermal equivalents must be applied consecutively, in order to generate suitable nutritional and immunological environment before the application of epidermal layer. A large variety of acellularized dermal substitutes mainly produced from allogeneic, xenogeneic dermal matrices (e.g. AlloDerm,[108] GraftJacket,[109] and Matriderm[110]) or synthetic materials (e.g. Integra,[111] Biobrane,[112] and Hyalomatrix PA[113]) could stimulate the ingrowth of autogeneic ECs and fibroblasts that help defected areas form dermal structure after transplantation, while some of the bioactive dermal substitutes consisting of human neonatal fibroblasts (e.g. Dermagraft[114] and TransCyte[115]) spontaneously show benefits in vascularization, epidermalization, as well as ECM formation.

### Double-layered substitutes

Double-layered skin substitutes combine both epidermal and dermal layers to histologically mimic the structure of normal skin. Without economic consideration, currently the epidermal/dermal composite TES (e.g. Apligraf[116]) represents the best treatment for skin repair, when compared with single-layered products. In skin tissue engineering, the interaction between parenchyma and stroma appears to be instructive in programming tissue structure and function during epidermogenesis.[117] It is widely accepted that the inductive effects of fibroblasts on epithelial morphogenesis are mediated by cell-cell interactions and ECM secretion.[118,119] Due to the interplays between multiple seed cell types, epidermal/dermal substitutes show enhanced wound closure and keratinization capability. A full-thickness living skin analogue (Activskin[120]) developed by our team has been used to treat refractory ulcers successfully in clinical applications. The treatment of refractory ulcers has drawn greater attention in recent decades owing to the increase in life expectancy in the industrialized world and the associated increase in the prevalence of comorbid conditions such as diabetes and vascular disease.[121]

### More complicated substitutes

In addition to the clinically available TES, great efforts have been made exploring optimized 3D constructs to repair skin tissue. Recent advances in skin biology have stressed the importance of cell-cell interactions during epidermal morphogenesis. In particular, progress in the field of skin tissue engineering has contributed tremendously to our knowledge about *in vitro* epidermal morphogenesis, resulting in the reconstruction of highly sophisticated and innovative 3D skin equivalents that mimic human skin in terms of tissue architecture and function, including hair follicle,[122] capillary network,[59,62] sensory innervation,[123] adipose tissue,[124] and pigment production.[30,125] When grown in the environment that mimics the anatomical position of skin, these bioengineered skins present definite differences in epidermal regeneration according to the types of seed cells used in the dermal substitutes. An exhilarating advancement is the elucidation of the crucial role of MSCs in skin regeneration. Apart from the morphogenesis potential, recent experimental evidence also demonstrates that BMSCs combined with epidermal stem cells could accelerate wound re-epithelialization and have better therapeutic potential in activating blood vessel and hair follicle formation than epidermal stem cells alone.[126] Like BMSCs, ADSCs are capable of differentiating into various skin cells which subsequently contribute to the wound healing.[127] The result obtained in our recent study in a bilayered TES system shows that a mixture of dermal fibroblasts and ADSCs in a ratio of 1:1 is superior to fibroblasts or ADSCs alone in promoting keratinocyte proliferation and differentiation.[128] With the steps into wound healing echanisms, proper combinations of cells and their interplays in initiating healing may be a way out for permanent TES construction.

## Challenges and perspectives

Currently, most of the TES could only function as temporary substitutions in skin wound healing. Lack of immediate blood supply and activation of immune rejection are the two major problems preventing the permanent integration of allogeneic TES. Evidences have shown that most of the cells contained in TES do not survive for 1 month after transplantation.[129] This temporary nature, whether the substitutes are degradable or have to be removed, makes TES predominantly serve as “coverings” instead of a real organic regenerator. However, it is reasonable to believe that with the development of skin biology, material science, and engineering technology, the TES will finally have an equivalent structural and functional therapeutic outcome as autogeneic skin transplantation does.
